# Genital Human Papillomavirus Infection among Women in Bangladesh: Findings from a Population-Based Survey

**DOI:** 10.1371/journal.pone.0107675

**Published:** 2014-10-01

**Authors:** Quamrun Nahar, Farhana Sultana, Anadil Alam, Jessica Yasmine Islam, Mustafizur Rahman, Fatema Khatun, Nazmul Alam, Sushil Kanta Dasgupta, Lena Marions, Ashrafunnessa, Mohammed Kamal, Alejandro Cravioto, Laura Reichenbach

**Affiliations:** 1 Centre for Population, Urbanization and Climate Change, International Centre for Diarrhoeal Disease Research, Bangladesh (icddr,b), Mohakhali, Dhaka, Bangladesh; 2 Centre for Epidemiology and Biostatistics, Melbourne School of Population and Global Health, The University of Melbourne, Parkville, Australia; 3 Vanderbilt Institute for Global Health, Vanderbilt University School of Medicine, Nashville, Tennessee, United States of America; 4 Department of Social and Preventive Medicine, University of Montreal, Montreal, QC, Canada; 5 Department of Women’s and Children’s Health, Karolinska Institutet, Stockholm, Sweden; 6 Department of Obstetrics and Gynecology, Bangabandhu Sheikh Mujib Medical University, Shahbag, Dhaka, Bangladesh; 7 Department of Pathology, Bangabandhu Sheikh Mujib Medical University, Shahbag, Dhaka, Bangladesh; 8 International Vaccine Institute, SNU Research Park, Gwanak-gu, Seoul, Republic of Korea; 9 Population Council, Washington, DC, United States of America; Georgetown University, United States of America

## Abstract

**Background:**

There has been no population-based study on human papillomavirus (HPV) prevalence or its genotypes in Bangladesh; a country eligible for GAVI funding for HPV vaccine.

**Methods:**

We used baseline survey data of a prospective cohort study that was conducted in one urban and one rural area of Bangladesh. A total of 997 urban and 905 rural married women, aged 13 to 64 years, were enrolled in the baseline during July-December, 2011. Information was collected on socio-demographic characteristics and potential risk factors for HPV infection followed by gynecological examination and collection of endocervical samples using the cervical cytobrush (Digene cervical sampler). HPV DNA testing was done by Polymerase Chain Reaction (PCR) using a consensus primer set.

**Results:**

Prevalence of any HPV infection was 7.7% with no significant difference between urban and rural women. Most common high-risk genotypes were HPV16, HPV66, HPV18, HPV45, HPV31 and HPV53. Urban women working as housemaids or garment workers were at higher risk of any HPV infection (OR = 2.15, 95% CI: 1.13–4.11) compared to housewives. Rural women whose husband lived overseas were almost two times more likely to have any HPV infection (OR = 1.93; 95% CI 1.05–3.55) compared to women whose husbands lived with them.

**Conclusion:**

The prevalence of HPV infection among Bangladeshi women is similar to other regions of Asia. However, type-specific patterns are different. The study findings will inform the formulation of HPV vaccination policies in Bangladesh, monitoring the impact of vaccination programmes, and the identification of target populations for screening.

## Introduction

Cervical cancer is a major public health problem worldwide. It is the fourth most common cancer in women with an estimated 527,624 new cases and 265,653 deaths in 2012 [1]. Around 85% of these new cases and 86% of deaths occur in less developed countries [2]. In Bangladesh, cervical cancer is the second most common cancer among females with an estimated 11,956 new cases and 6,582 deaths in 2012 [3].

Human papillomavirus (HPV) is one of the most commonly acquired sexually transmitted infections (STIs) and a significant source of morbidity and mortality [4], [5]. Persistent infection with certain types of HPV is a necessary cause of cervical cancer [6]. The World Health Organization has defined 12 HPV types (16, 18, 31, 33, 35, 39, 45, 51, 52, 56, 58 and 59), as high-risk (HR) or cancer-causing types and some other subtypes (68, 73, 26, 30, 53, 66, 67, 69, 82 and 85) as possible cancer-causing types [7]. Of these, HPV16 and HPV18 account for at least 71% of all cases of cervical cancer worldwide [8]. The other types (6, 11, 13, 40, 42, 43, 44, 54, 61, 70, 72, 81 and 89) that are mostly linked to genital warts are classified as “low-risk” (LR) types [9].

The prevalence of HPV infection has shown to vary by region, country and within a country by population sub-groups [10]. While the estimated global HPV prevalence is reported to be 11.7%, country specific prevalence ranges between 1.6% and 41.9% [11]. HPV prevalence is higher (14.3%) in developing regions than developed regions (10.3%). Type-specific distribution of HPV infection also varies by geographic region [12], [13]. Some HPV types are more prevalent in the Asia Pacific region than other regions [14].

The literature identifies several risk factors for the acquisition and persistence of HPV infection. Age is a strong predictor, although there are notable differences in age curves of HPV infection across regions [15]–[17] Other factors include number of recent/lifetime sexual partners, age at onset of sexual activity, socioeconomic status, male circumcision, condom use, oral contraceptive use, smoking [4], [18]–[25], use of public bathhouses [26] and low education [27]. High parity was also identified as a risk factor for HR-HPV infection [28].

While some epidemiological research on HPV has been conducted in the South Asian region [29], only a few studies have reported HPV prevalence among selected populations in Bangladesh such as female sex workers [30] and cancer patients in tertiary level hospitals [31], [32]. Moreover, there have been no systematic population-based studies to estimate HPV prevalence or risk factors for HPV infection in Bangladesh [33]. Previous systematic reviews have postulated the prevalence of HPV in South Asian countries and region to be lower than other areas of the world [10]. Similarly, we hypothesized HPV prevalence to be similar to those found in India and other neighboring countries. In this paper, we report the results from a baseline survey of a population-based prospective cohort study of the type-specific and age-specific distribution of HPV infection and its risk factors among ever-married women in an urban and a rural area of Bangladesh.

## Materials and Methods

### Study Population and Sampling

**Figure 1:**
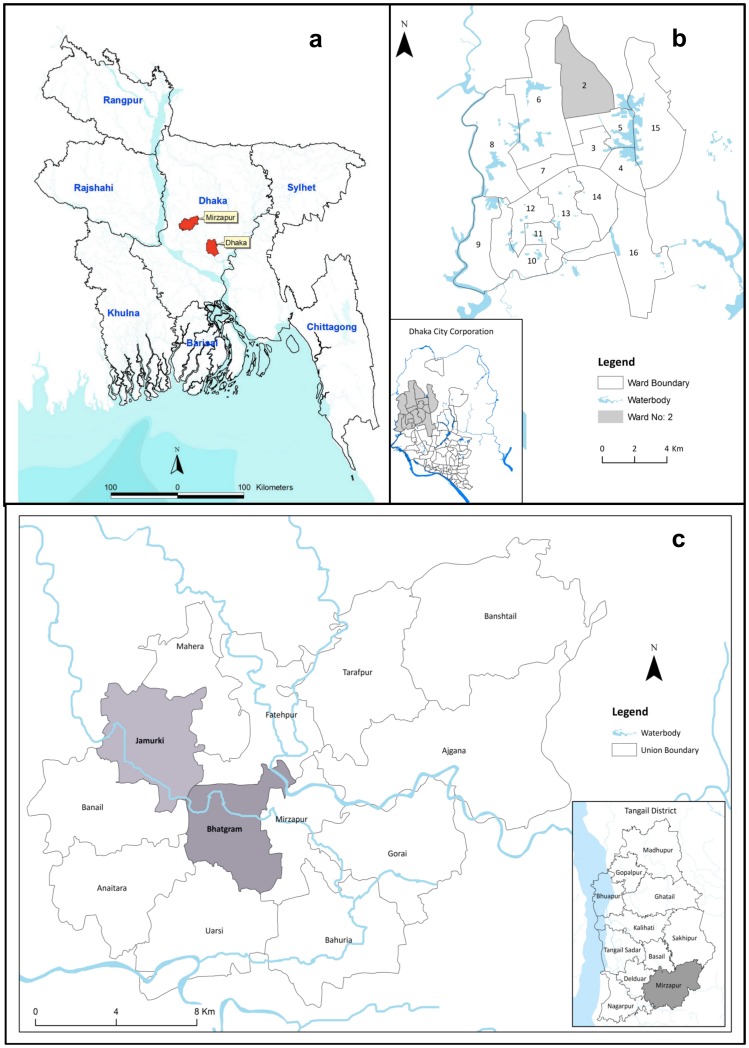
Map of Bangladesh showing (a) Dhaka and Mirzapur (b) Dhaka City Corporation showing Ward 2 and (c) Tangail District showing Mirzapur sub-district and 2 selected unions.

The baseline survey was conducted between July and December 2011 and was implemented in two areas of Bangladesh; one rural and one urban (Figure 1a). The urban area is located in Dhaka, the capital of Bangladesh, which is the largest metropolitan city in the country. Dhaka city is divided into 92 administrative wards (smallest administrative urban geographic unit), each with an approximate population of 90,000 or more. The International Centre for Diarrhoeal Disease Research, Bangladesh (icddr,b), an international research organization, has maintained a surveillance site for one of its on-going research projects since 2010 in Ward 2 (Figure 1b) covering a population of approximately 27,000. For the purpose of surveillance, Ward 2 is divided into 9 clusters. For this study, we purposely selected 3 of these 9 clusters. All ever-married women aged 13–64 years and living in these three clusters (n = 4,484) constituted the sampling frame for the urban component of the study.

The rural site of the study is located in Mirzapur, a sub-district of Tangail district, which is 56 kilometers northwest of Dhaka. In Mirzapur, icddr,b has been running a demographic surveillance since 2007 covering a population of approximately 240,000 in eight unions (smallest administrative rural geographic unit comprising of mauzas and villages and having union parishad institution). We purposely selected two of the eight unions (Figure 1c) that comprised a total of 35 villages and then randomly selected eight of these villages. All ever-married women aged 13–64 years (n = 5,222) from these eight villages constituted the sampling frame for the rural site.

Using systematic random sampling, every third woman in the sampling frame in both urban and rural areas was approached and asked to participate in the study. In the field, the selected women were identified by their name and address recorded in the listing database (urban site) or in the surveillance data base (rural). In both the urban and rural areas, the clusters and villages under study were purposely selected for feasibility issues considering proximity to icddr,b’s field offices, logistics and cost-related issues.

#### Ethics Statement

The study protocol was approved by the Ethical Review Committee (ERC) of icddr,b. Informed written consent was taken from women aged 18–64 years whereas informed written assent was taken from women aged 13–17 years. For the later group, the assent form was read out to these women in the presence of their parents/legal guardians. If agreed, the assent form was then signed by the participants in the presence of their parents/guardians. The parents/guardians signed the same form.

### Data Collection

In both study areas, women were first approached at their home and eligibility was determined by trained female research staff. They were briefed about the study background and purpose, and the potential roles they would have in the study. Women who were eligible and willing to participate were sent to a study field office for interview, clinical examination and sample collection. In the case of a refusal, the next eligible woman on the list was approached.

Upon arrival at the study field office, a female study physician provided them with detailed information about the study process including the process for collecting the cervical sample. On agreeing to participate in the study, the women were further screened for eligibility criteria by the physician; upon meeting these criteria they were then taken to the next room for interview. Women who were pregnant, or post-partum, or with a history of hysterectomy, cervical cancer or uterine prolapse were excluded from the study. Women with major psychological/psychiatric problems were also excluded.

After informed, voluntary, written consent or assent, six trained female research assistants collected information on socio-demographic characteristics (age, education, occupation, marital status, age at marriage, husband’s residency, and monthly household expenditure), sexual and reproductive history (parity, number of life-time sex partners, number of husband’s life-time sex partners) and other risk factors, such as use of oral contraceptives, tobacco and condom use. Two trained female research physicians performed clinical examinations, including visual examination of the vulva. Following this, the speculum was inserted and the vagina and cervix were examined for any discharge, erosion or lesions. The physician then collected cervical samples using the cervical cytobrush (Rovers, Cervex-Brush, Oss, The Netherlands). Following sample collection slides for cervical cytology were prepared The head of the brush was then detached in 10 ml BD SurePath (BD Diagnostics, Burlington, NC, USA) and stored at 4°C. The specimens (including slides) were then transported to the virology laboratory of icddr,b maintaining appropriate temperature and stored at 4°C until tested. While HPV DNA testing was performed at icddr,b, the cervical slides were sent to the pathology department of Bangabandhu Sheikh Mujib Medical University (BSMMU) for testing. The results of both the HPV DNA test and cervical cytology were provided to the study participants within 2–4 weeks of sample collection. Women presenting the possibility of precancerous or cancerous changes were referred to BSMMU for further assessment and treatment. The details of cervical cytology will be published elsewhere.

### HPV Testing

#### Sample processing and DNA extraction

A 6ml aliquot of cervical swab sample was centrifuged at 5000rpm for 15 min. After decanting the supernatant, the pellet was resuspended in 500 µl phosphate buffer saline. DNA extraction was carried out with a 200 µl aliquot of this concentrated sample using a DNeasy Blood and Tissue Kit (Qiagen, Hilden, Germany) according to the manufacturer’s instructions. The extracted DNA was stored at −20°C until tested.

HPV genotyping was carried out in three steps: 1) Detection of HPV L1 gene using PCR; 2) Genotyping using nucleotide sequencing, and 3) Identification of co-infection and genotypes of a small number of HPV positive cases for which typing could not be done in step 2.

#### Detection of HPV L1 gene using PCR

The first round of PCR amplification was carried out in a 25 µl reaction volume containing 10 µl extracted DNA as a template, 2.5 µl Qiagen 10× PCR buffer, 0.25 µl Hotstar Taq polymerase (Qiagen, Hilden, Germany) (5 U/µl), and 20 pmol papillomavirus specific consensus primers MY09/MY11 [34] and 10 mM of each dNTPs. This reaction mixture was preheated at 95°C for 15 min for polymerase activation and amplified for 40 cycles under the following conditions: denaturation at 94°C for 20 s, annealing at 55°C for 20 s and extension at 72°C for 40 s, plus a final extension step at 72°C for 7 min. One microlitre from the first PCR was further amplified with the inner pair of primers GP5+/GP6+ [35] in a 25 µl reaction mixture. The second round of amplification was performed as follows: 94°C for 60 s, 60°C for 60 s and 72°C for 60 s, plus an additional final extension step at 72°C for 5 min.

#### Genotyping using nucleotide sequencing

HPV positive PCR products either visible in the first or second PCR round were sequenced using respective PCR-product primers with a Big-Dye Xterminator ready reaction kit v3.1 (Applied Biosystems, Foster City, CA. USA) in an ABI 3500xL genetic analyser (Applied Biosystems, Foster City, ca. USA). Genotyping of HPV was inferred by comparing the resulting sequence for each sample to the GenBank database using BLAST (Basic Local Alignment Search Tool). This method identified HPV types in all samples except six.

#### Identification of co-infections

To identify co-infections, all HPV positive samples were reassessed with a commercially available real time PCR kit (Hybribio HPV Detection Kit, Hybribio Ltd, Wanchai, Hong Kong). The test specifically identifies HPV16 and HPV18 while concurrently detecting the rest of the 10 high risk types (31,33,35,39,45,51,52,56,58,59) and two possible high-risk type (66, 68) in a pooled result (other HR-HPV other than 16/18) and any of the 5 LR-HPV types (6,11,42,43,44). The reassessment also assisted us to identify genotypes of six HPV positive cases for which typing could not be done using PCR sequencing in step 2.

To verify the presence of human genomic DNA in samples, a 268 bp fragment of the β-globin housekeeping gene was amplified using primers GH20 (5′-CAACTTCATCCACGTTCACC-3′), PC04 (5′-GAAGAGCCAAGGACAG GTAC-3′) and HMBB01 (5′-GCGACCCAATGCAAATTGGT-3′) [36]. This process was critical to verifying the presence of adequate cervical epithelial cells in the sample to be tested for HPV.

### Statistical Analysis

Descriptive analyses were performed to compare women in rural and urban areas by their socio-demographic group, reproductive history and other variables such as age, education, occupation, parity, number of sexual partners, use of oral contraceptives, use of condoms and use of tobacco. Test of significance was performed using Chi-square or Fisher’s test with a 5% level of significance. The prevalence of “any HPV” infection was calculated as the proportion of women testing positive for any type of HPV infection. Frequency distribution of type-specific information of HR-HPV positive cases was also collated. In the calculation of prevalence for HR-HPV infection, women who were co-infected with HR-HPV and LR-HPV were categorized as HR-HPV positive cases and included in the calculation of HR-HPV type prevalence. Age-specific distribution of any HPV infection, LR-HPV and HR-HPV infections were calculated separately for urban and rural women.

Two forward step-wise multivariate logistic regression models were generated separately for urban and rural women to estimate adjusted odds ratios with 95% confidence intervals using “Any HPV infection” and “HR-HPV infection” as dependent variables. In the first model with “Any HPV infection” as the dependent variable, all women tested positive for any HPV infection were coded as “1” and the rest were coded as “0”. Similarly, in the second model, all women who tested positive for HR-HPV infection, either single or co-infection, were coded as “1” and the rest were coded as “0”. Due to the sampling design and possible effect modification, data analyses were carried out separately for urban and rural samples. Statistical analyses were performed using SPSS, version 17.0 and STATA/SE 12.0.

## Results

In the urban area, a total of 1,855 eligible women were approached of which 1,152 (62%) agreed to participate; interviews were completed and samples collected for 1,113 (97%) women. Out of these, 997 women were currently married and having an active sexual relationship with their husband. Similarly, in the rural area, a total of 1,499 eligible women were approached, of which 1,159 (77%) agreed to participate. Interviews were completed and samples collected from 924 (80%) women, of whom 905 women were currently married and having an active sexual relationship with their husband. The rest of the women who were approached but not included in this study either refused to take part (n = 597; 18%), did not show up in the project office on the scheduled day of interview (n = 330; 10%), or were found ineligible after examination by the study physicians (n = 390; 12%). Of the women who were interviewed and from whom samples were collected, some were either divorced, widowed or separated at the time of the interview (n = 135; 7%) and information on their husband’s residence or condom use was not appropriate to collect and therefore, these women were excluded from the analysis.

### Background characteristics

Table 1 shows background characteristics of women in the study. More than half of the women were between 25 and 44 years of age with no significant difference in mean age between urban and rural women. More urban women than rural women had completed secondary education or higher (31% versus 12%), were working (29% versus 7%; p<0.01) and had a median monthly household expenditure (16,872 versus 8,790 Bangladeshi taka [BDT]; 1 US$ = 78 BDT; p = 0.000). A significant percentage of urban women worked as garment workers or housemaids (14%) compared to rural women (<1%; p<0.001). Urban women also had a higher mean age at marriage compared to rural women (17.4 years versus 16.0 years; p = 0.000).

**Table 1:** Background characteristics of study sample, by sites.

	Urban (n = 997)		Rural (n = 905)		
Characteristics	No.	(%)	No.	(%)	P Value
**Age (in years)**					
13–24	231	(23.7)	193	(21.3)	0.000
25–34	415	(41.6)	322	(35.5)	
35–44	213	(21.3)	271	(29.9)	
45 –64	138	(13.8)	119	(13.1)	
Mean age in years		32.02		32.95	
**Education**					
No education	155	(15.5)	266	(29.3)	
Primary	532	(53.3)	532	(58.7)	0.000
Secondary or more	310	(31.0)	107	(11.8)	
Mean years of school completed		6.93		4.98	0.000
**Occupation**					
Housewife	709	(71.1)	842	(93.0)	
Garments worker/Housemaid	139	(13.9)	6	(0.6)	0.000
Other work*	149	(14.9)	57	(6.3)	
**Monthly household expenditure in BDT (1** **US$ = 78 BDT)**					
<10,000	207	(20.7)	533	(58.9)	0.000
10,000–19,999	473	(47.4)	312	(34.4)	
20,000+	317	(31.8)	60	(6.6)	
Median income		16,872		8,790	0.000
**Age at marriage (in years)**					
= <17	553	(55.4)	651	(71.9)	0.000
18–20	293	(29.3)	203	(22.4)	
20+	151	(15.1)	51	(5.6)	
Mean age at marriage		17.34		16.04	0.000
**Husband’s residency**					
Always stays at home	874	(87.6)	669	(73.9)	
Sometime spend night outside	68	(6.8)	72	(7.9)	0.000
Currently overseas	55	(5.5)	164	(18.1)	
**Parity**					
0	67	(6.7)	63	(6.9)	
1–2	413	(41.4)	387	(42.6)	0.789
3+	517	(51.8)	455	(50.2)	
Mean number of children born		2.94		2.12	0.522
**Woman’s lifetime sex partner**					
One	938	(93.7)	875	(96.6)	0.003
More than one	62	(6.2)	30	(3.3)	
**Husband’s lifetime sex partner**					
One	784	(78.6)	730	(80.6)	0.273
More than one	213	(21.3)	175	(19.3)	
**Oral contraceptive use**					
Never	184	(18.5)	120	(13.2)	0.002
Ever	813	(81.5)	785	(86.7)	
**Condom use**					
Never	422	(42.3)	604	(66.7)	0.000
Ever	574	(57.6)	301	(33.2)	
**Tobacco use (including chewing)**					
Never	752	(75.4)	676	(74.7)	0.713
Ever	245	(24.5)	229	(25.3)	

*Includes government and private service, business, daily wager, agriculture, tailor, poultry, handicrafts and tutor.

**Bangladeshi Taka (BDT); 1 US$ = 78 BDT.

While the majority of women resided with their husbands in both areas (88% of urban and 74% of rural women), a higher proportion of rural women had a husband living overseas (18%) compared to urban women (6%). About half of the women had three or more children with no difference in the mean number of children between urban and rural women. Most women in both areas reported one lifetime sex partner. While the majority of women reported their husband to have one lifetime sex partner, one in five women reported their husband as having more than one lifetime sex partner, with no difference between urban and rural women. Ever use of oral contraceptives was slightly higher among rural women compared to urban women (87% versus 82%; p = 0.002); whereas ever use of condoms was significantly higher among urban women compared to rural women (58% versus 33%; p = 0.000).

### Prevalence of HPV infection

**Figure 2:**
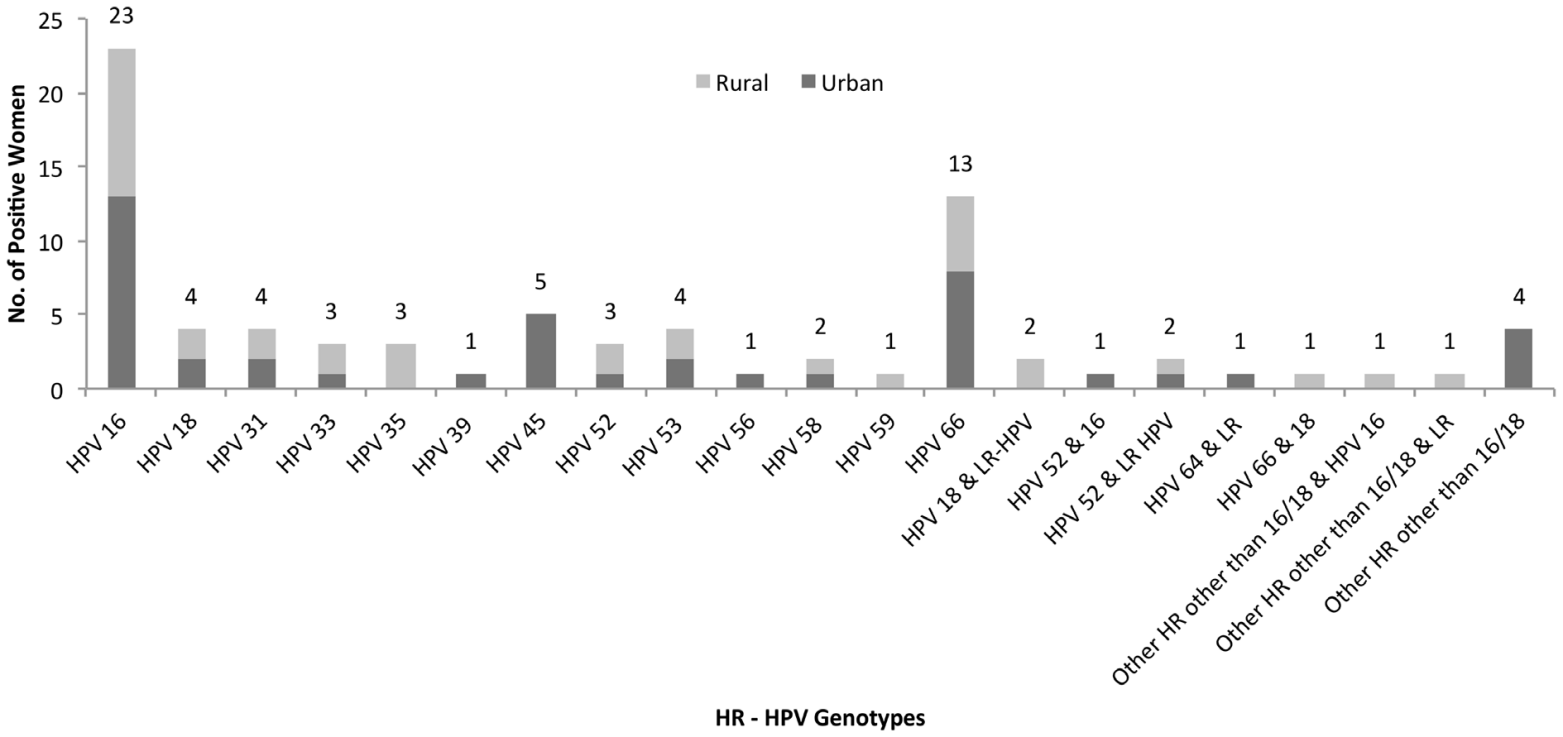
Frequency distribution of HR-HPV genotypes in women in Bangladesh (n = 80) by site. Number of HR-HPV positive women is shown along the Y-axis and HR-HPV genotypes are shown along the X-axis.

Overall, the prevalence of any HPV infection was 7.7% with no significant difference between urban and rural women (7.9% and 7.5% respectively; p = 0.87). A total of 93 women had single-type HPV infection (4.9% overall and 64.1% of HPV positive women) and 52 women had multiple HPV infection (2.7% overall and 35.9% of HPV positive women). HR-HPV infections, single and co-infection combined, were more common (4.2%) than LR-HPV infections (3.4%) with no significant variation between urban and rural women. The most commonly found HR-HPV types in either single or co-infection were HPV16, HPV66, HPV18, HPV45, HPV31 and HPV53. Other HR-HPV types included HPV33, HPV35, HPV52 and HPV58 (Figure 2).

**Figure 3:**
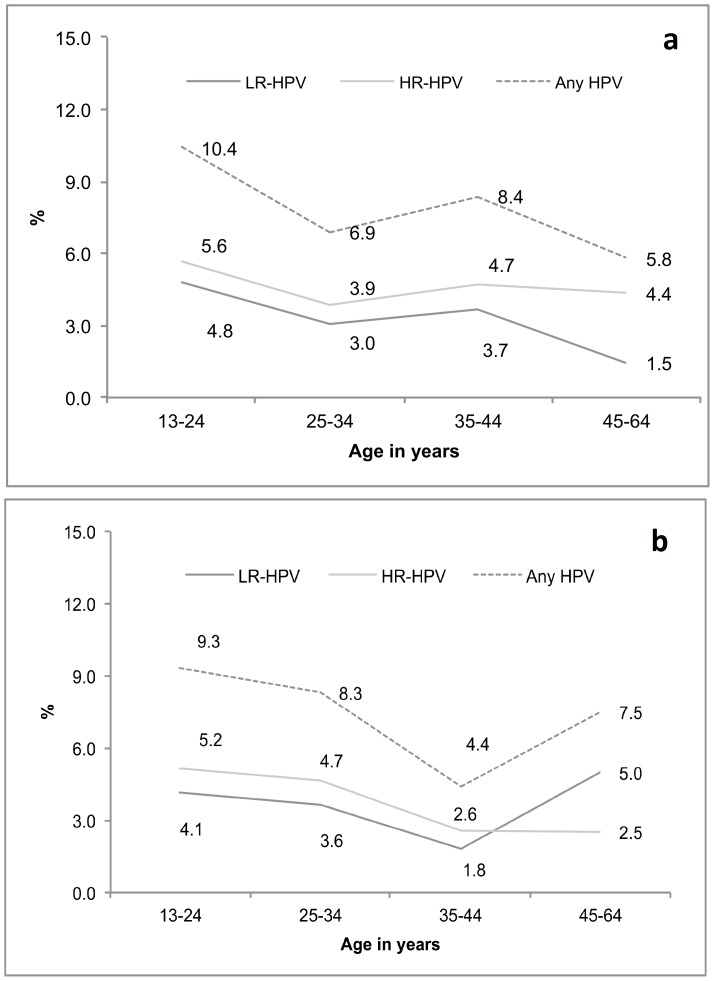
Age-specific prevalence of HPV infection by (a) Urban and (b) Rural study populations.

Age-specific prevalence of any HPV infection and infection by HR and LR-HPV types are shown in Figure 3a and b. Age-specific prevalence of any HPV infection was slightly different for urban and rural women. For urban women, age-specific prevalence decreased from 10.4% for 24 years of age or younger to 6.9% for 25–34 years of age; thereafter, prevalence increased to 8.4% at 35–44 years and then decreased again to 5.8% for women 45 years and older (Figure 3a). Infection by HR types dominated LR types for urban women in all age groups; however, the differences were most marked in the oldest age group.

A slightly different HPV infection pattern was observed for rural women. Age-specific prevalence decreased from 9.3% at 24 years or below to 8.3% and 4.4% respectively for the next two age groups of 25–34 and 35–44 years; thereafter, it increased to 7.5% for women aged 45 years and older (Figure 3b). When stratified by type of HPV infection, we found HR infection to decrease with age and to be relatively stable after 35 years, while LR infection decreased with age until 35–44 years and thereafter, increased in older women (45 years and older).

### Risk factors for HPV infection

Table 2 presents results from multivariate logistic regression models of risk factors associated with any HPV infection and those associated with HR-HPV infection. After controlling the effects of other factors in the model, we did not find any effect of age on any HPV infection or HR-HPV infection. Education was found to have a varied effect on HPV infection by study site. While no effect of education was seen on HPV infection (any or HR-HPV) in urban women, having primary, secondary or higher education significantly decreased the risk of any HPV infection in rural women (OR = 0.46, 95% CI 0.23–0.92; OR = 0.34, 95% CI 0.12–0.98 respectively) when compared to those who had no education. The risk of getting HR-HPV infection was even lower for these women if they had primary education (OR = 0.34, 95% CI 0.14–0.83); however, no effect of completing secondary or higher education was seen on HR-HPV infection in rural women.

**Table 2:** Multivariate logistic regression analyses showing the factors affecting HPV infection by study sites.

Covariates	Urban		Rural	
	aOR* for any HPVinfection (95% CI)	aOR for HR-HPVinfection (95% CI)	aOR for any HPVinfection (95% CI)	aOR for HR-HPVinfection (95% CI)
**Age (in years)**				
13–24 (R)	1	1	1	1
25–34	0.54 (0.27–1.05)	0.50 (0.20–1.21)	1.27 (0.62–2.59)	1.31 (0.59–3.39)
35–44	0.58 (0.24–1.39)	0.49 (0.16–1.52)	0.65 (0.24–1.80)	0.70 (0.18–2.68)
45–64	0.40 (0.14–1.18)	0.53 (0.14–1.96)	1.21 (0.348–3.81)	0.70 (0.13–3.83)
**Women’s Education**				
No education (R)	1	1	1	1
Primary	1.00 (0.48–2.08)	1.14 (0.42–3.10)	0.46 (0.23–0.92)	0.34 (0.14–0.83)
Secondary+	1.91 (0.82–4.43)	2.43 (0.78–7.55)	0.34 (0.12–0.98)	0.33 (0.08–1.31)
**Women’s Occupation**				
Housewife (R )	1	1	a	a
Garment worker/housemaid	2.15 (1.13–4.11)	4.11 (1.80–9.40)	a	a
Other work	0.56 (0.24–1.30)	0.65 (0.21–1.93)	a	a
**Monthly household expenditure** **in BDT (1US$ = 78 BDT)**				
<10,000 (R)	1	1	1	1
10,000–19,999	0.96 (0.51–1.79)	1.45 (0.60–3.52)	1.44 (0.83–2.50)	0.40 (0.16–1.03)
20,000+	0.91 (0.42–1.97)	1.99 (0.69–5.66)	1.83 (0.64–5.23)	1.98 (0.59–6.59)
**Parity**				
0 (R)	1	1	1	1
1–2	0.91 (0.36–2.30)	1.36 (0.36–5.14)	0.47 (0.20–1.11)	0.49 (0.15–1.55)
3+	1.41 (0.50–3.95)	2.19 (0.51–9.38)	0.28 (0.09–0.80)	0.31 (0.07–1.27)
**Husband’s residency status**				
Always stays home (R)	1	1	1	1
Stays outside sometime	0.67 (0.24–1.83)	0.43 (0.09–1.94)	1.17 (0.44–3.15)	0.85 (0.18–3.87)
Stays overseas	0.46 (0.10–1.98)	0.73 (0.16–3.20)	1.93 (1.05–3.55**)**	1.77 (0.78–3.97)
**Women’s life time sex-partner**				
1 (R)	1	1	a	a
2 or more	3.80 (1.79–8.08)	2.75 (0.99–7.59)	a	a
**Husband’s Life time sex-partner**				
1 (R)	1	1	1	1
Two or more	1.47 (0.83–2.61)	1.94 (0.94–4.00)	0.50 (0.22–1.16)	0.23 (0.05–1.08)
**Use of oral contraceptive**				
Never (R)	1	1	1	1
Ever	0.99 (0.52–1.86)	0.73 (0.34–1.57)	2.03 (0.80–5.12)	2.06 (0.57–7.40)
**Use of Condom**				
Never (R)	1	1	1	1
Ever	1.29 (0.77–2.16)	1.45 (0.74–2.87)	0.62 (0.33–1.15)	0.48 (0.19–1.19)
**Use of Tobacco**				
Never (R)	1	1	1	1
Ever	1.27 (0.74–2.17)	1.07 (0.53–2.19)	1.12 (0.56–2.39)	0.71 (0.25–2.02)

a = not enough sample aOR = adjusted Odds Ratio: adjusted for variables in the table.

Abbreviations: HR – HPV = High Risk Papillomavirus; OR = Odds Ratio; CI = Confidence Intervals; (R) = Reference group.

Occupation had the strongest effect on HPV infection for urban women. Urban women who were working as housemaids or garment workers were at higher risk of any HPV infection (OR = 2.15, 95% CI: 1.13–4.11) compared to those who reported being housewives. The risk doubled for infection with HR-HPV subtypes (OR = 4.11, 95% CI: 1.80–9.40) for this group of women.

Parity was found to be associated with any HPV infection for rural women only. In particular, having three or more children lowered the risk of any HPV infection for rural women (OR = 0.28; 95% CI 0.09–0.80). Similarly, husband’s residency status was found to be associated with any HPV infection for rural women. Rural women whose husband lived overseas were almost twice as likely to have any HPV infection (OR = 1.93; 95% CI 1.05–3.55) compared to those women whose husbands lived with them.

The number of lifetime sex partners of urban women was significantly associated with detection of any HPV infection; women reporting two or more partners were almost four times more likely to have any HPV infection compared to those reporting only one sex partner (OR = 3.80, 95% CI: 1.79–8.08). We did not find any association between the numbers of lifetime sex partners of women with HR-HPV infection. We also did not find any association between the number of the husband’s sex partners with the risk of any HPV infection or HR-HPV infection. No effect of contraceptives or tobacco use was found in our analysis. We did not have enough rural samples to investigate the effect of occupation or number of sexual partners on HPV status.

## Discussion

To the best of our knowledge, this is the first study to investigate the prevalence of HPV infection and its type-specific distribution in population-based samples of urban and rural women in Bangladesh. The overall prevalence of any HPV infection was found to be 7.7% in this study. This is similar to the prevalence reported in other Asian countries –9% in Nepal [37], 6.7% in China [38] 9.1% in Thailand [39] and 11.4% in Indonesia [40].

HR-HPV was more common than LR-HPV infection in almost all age groups, except among rural women older than 44 years. HPV16 was the most frequently detected type, as found in other parts of the world, including Asia [14]. However, the other HR types found in our study were somewhat different than the types found among female sex workers in Bangladesh [33] and in other Asian countries. HPV66 was the second most common type followed by HPV18 in our study, while HPV18 followed by HPV33 were most commonly found among female sex workers [33]. HPV58 was the second most common HR type reported in other Asian countries [14]. Other prevalent types in the region are HPV18, HPV31, HPV33, HPV35, HPV39, HPV45, HPV51, HPV52 and HPV56 [14] compared to HPV18, HPV45, HPV31 and HPV53 in our study.

Similar to other countries, we found HPV infection to be most common in younger women with the peak prevalence occurring in women younger than 25 years of age; prevalence started to decline after 30 years of age. We found a secondary increase after age 44 for rural women and ages 35–44 for urban women. In Bangladesh girls marry at an early age and the study was conducted among married women. This may explain high HPV infection in the younger age group. However, the secondary peak seen during 35–44 years in urban women and 45–64 years for rural women requires further explanation.

Bangladesh has made remarkable progress in terms of population, economic and health indicators in recent years [41]. There are several factors that indicate an improvement in Bangladesh’s social sector, such as rising educational levels, particularly in women [42]–[44], and increased economic opportunities, especially in urban areas. The garment industry accounts for approximately 80% of the country’s total exports and roughly 85% of the workers in the garment industry are women. For women, this has led to increased employment and empowerment but also exposure to changing social dynamics and environments that may increase their engagement in intimate relationships [45] and sexual exploitation [46], [47], thereby increasing their likelihood of HPV infection. This may explain the significant effect of women’s occupation on HPV infection in the urban setting of this study.

Occupation was also a significant predictor for HPV infection in the rural study site. In this case however, it was the risk posed by the occupation of women’s husbands. Economic opportunities outside of Bangladesh draw rural men to leave their families for long periods of time for work overseas. This may increase their opportunity for engaging in risky sexual behaviors, thus increasing their wives vulnerability to HPV infection. All these factors may help to explain the secondary peak of HPV infection in our study in women in their late 30′s and 40′s.

While we found a similar prevalence of any HPV infection and HR-HPV infections among rural and urban women, we found some important differences in risk factors for infection between rural and urban women. Education had a protective effect for rural women, but not for urban women. Urban women working as garment workers or housemaids were at an increased risk of HPV infection. For urban women, the number of sexual partners was highly associated with HPV infection, a finding consistent with other settings [48]. This finding is also in agreement with that from a previous study conducted in Bangladesh where duration of sex work as interpreted as increased number of sex partners was found to be a strong predictor for HPV infection [33].

Another common economic opportunity for women in urban areas is to work as domestic servants or housemaids. This occupation may also expose women to increased risk of sexual exploitation as supported by other studies [49], [50]. It is important to note that occupation is a significant risk factor for urban women in our study. This may suggest the importance of using garment factories as entry points for HPV education and screening programmes for cervical cancer.

We did not find any association between oral contraceptive use with HPV infection as found in other studies [48], [51]. This lack of association may be partially explained by the fact that we collected information on use at any point in the women’s life-time (ever-use) and not current use or duration of use. Similarly, our analysis did not demonstrate the beneficial effect of condom use in protecting against HPV infection as shown in other studies [52], [53]. This may be due to the very low rates of condom use in the country [54]. There was also a lack of association between tobacco use and HPV infection, which is again contrary to the current knowledge that smoking increases the risk of HPV infection [5], [51]. Very low use of tobacco among the study population may explain this finding. The lack of association between condom use and tobacco consumption and HPV infection needs further investigation.

Our study used population-based socio-economically diverse samples from two existing surveillance systems. However, limited participation (62% of sample in urban and 77% of rural sample) may raise questions about the representativeness of the study findings. To address this, we examined the basic socio-demographic characteristics of those participating in the study and those who did not, and we found the two groups to be similar, suggesting limited plausibility for selection bias.

Our study adds immense value to the existing knowledge of the burden of HPV infection among the selected urban and rural female population in Bangladesh. The identification of genotypic distribution will help further understanding of the risk of developing cervical cancer in the study population. While we found HPV16 to be the predominant type, HPV66 was the second major HR-HPV type identified in our study population, followed by HPV18. Epidemiological evidence of the carcinogenicity of HPV66 was previously judged sufficient; however, HPV66 has rarely been identified in populations [55]. The currently licensed vaccines do not include HPV66 and it is unknown how these vaccines will perform against this type, which appears to be common among Bangladeshi women.

Given that HPV infection is a necessary cause of cervical cancer and preventing it is the most effective way to prevent cervical cancer. HPV vaccines have been proven to be an effective and safe measure to prevent HPV infection and disease related to the vaccine-specific genotypes in women with no evidence of past or current HPV infection [56], [57]. Recent population-based studies have confirmed these findings [58], [59]. Based on the results of these previously published studies, we can be certain that the introduction of a vaccine against HPV16 and HPV18 will reduce the bulk of cervical lesions in Bangladeshi women, as has been seen in other countries such as Australia [60].

This study provides the first population-based HPV prevalence data in Bangladesh. While the findings are similar to studies from neighboring countries, the type-specific distribution, as well as identification of risk factors will provide additional evidence to guide Bangladesh’s response through a cervical cancer prevention programme. The findings are especially timely as the Government of Bangladesh is planning to apply to the Global Alliance for Vaccines and Immunization (GAVI) for funding for pilot testing of the HPV vaccine in a peri-urban setting. The evidence from this study will provide necessary country-specific evidence for this application.
