# Correction: Genital Human Papillomavirus Infection among Women in Bangladesh: Findings from a Population-Based Survey

**DOI:** 10.1371/journal.pone.0114655

**Published:** 2014-11-20

**Authors:** The PLOS ONE Staff

There is an error in affiliation 1 for authors Quamrun Nahar, Farhana Sultana, Anadil Alam, Mustafizur Rahman, Fatema Khatun, Nazmul Alam and Sushil Kanta Dasgupta. Affiliation 1 should be: International Centre for Diarrhoeal Disease Research, Bangladesh (icddr,b), Mohakhali, Dhaka, Bangladesh.
